# Roadmap to strengthen global mental health systems to tackle the impact of the COVID-19 pandemic

**DOI:** 10.1186/s13033-020-00393-4

**Published:** 2020-07-29

**Authors:** Pallab K. Maulik, Graham Thornicroft, Shekhar Saxena

**Affiliations:** 1 George Institute for Global Health, 311-312 Elegance Tower, Jasola, New Delhi 110025 India; 2 University of New South Wales, Sydney, Australia; 3 Prasanna School of Public Health, Manipal, India; 4 Centre for Global Mental Health and Centre for Implementation Science, Institute of Psychiatry, Psychology and Neuroscience, King’s College London, London, UK; 5 Global Health and Population Harvard T H Chan School of Public Health, Boston, USA; 6 George Institute for Global Health, Sydney, Australia

**Keywords:** Mental health systems, COVID 19, Mental health services, Research, Mental health resources, Policy making

## Abstract

**Background:**

The COVID pandemic has been devastating for not only its direct impact on lives, physical health, socio-economic status of individuals, but also for its impact on mental health. Some individuals are affected psychologically more severely and will need additional care. However, the current health system is so fragmented and focused on caring for those infected that management of mental illness has been neglected. An integrated approach is needed to strengthen the health system, service providers and research to not only manage the current mental health problems related to COVID but develop robust strategies to overcome more long-term impact of the pandemic. A series of recommendations are outlined in this paper to help policy makers, service providers and other stakeholders, and research and research funders to strengthen existing mental health systems, develop new ones, and at the same time advance research to mitigate the mental health impact of COVID19. The recommendations refer to low, middle and high resource settings as capabilities vary greatly between countries and within countries.

**Discussion:**

The recommendations for policy makers are focused on strengthening leadership and governance, finance mechanisms, and developing programme and policies that especially include the most vulnerable populations. Service provision should focus on accessible and equitable evidence-based community care models commensurate with the existing mental health capacity to deliver care, train existing primary care staff to cater to increased mental health needs, implement prevention and promotion programmes tailored to local needs, and support civil societies and employers to address the increased burden of mental illness. Researchers and research funders should focus on research to develop robust information systems that can be enhanced further by linking with other data sources to run predictive models using artificial intelligence, understand neurobiological mechanisms and community-based interventions to address the pandemic driven mental health problems in an integrated manner and use innovative digital solutions.

**Conclusion:**

Urgent action is needed to strengthen mental health system in all settings. The recommendations outlined can be used as a guide to develop these further or identify new ones in relation to local needs.

## Background

The COVID-19 pandemic is not only affecting communities directly, but through its socio-economic consequences is also affecting the lives of many millions of families and individuals. The direct health impact, economic impact, and disruption of the social and community structures across the globe is potentiating a major international mental health crisis [1–4]. The mental health impacts of COVID-19 can be varied and severe and have been outlined recently [5]. The effect of this stress can vary from mild symptoms related to physiological or psychological functions such as sleep disturbance or low mood, mild stress for short periods of time that do not need any specific treatment and resolve when the primary stressors such as job loss or illness in family or poor social support are taken care off, to the more severe syndromal mental disorder which may need formal treatment from a mental health professional [6].

Anxiety, depression, increased alcohol and substance use, irritability, anger, insomnia and increased risk of suicide have been reported, as have been risk factors for mental disorders such as loneliness, domestic violence, physical violence. Individuals with existing mental disorders such as alcohol and substance use, cognitive impairment and dementia, childhood psychiatric disorders and adults needing long term follow up have been particularly affected due to lack of continued psychiatric care services and fragmentation of the existing health systems to provide adequate care. In addition, the direct impact of COVID 19 on mental illness of those infected or health workers involved in care of those infected is also significant, and is often precipitated due to increased stigma, social isolation and quarantine [5]. All this is even more complicated due to the socioeconomic impact of the pandemic on the lives of the poor and most disadvantaged communities such as homeless and migrant workers. The overall mental health impact of the pandemic is not transient but likely to continue for a long period even after the pandemic ends, as is evident from prior research on such severe epidemics [7, 8]. Researchers have highlighted the need for focussed research that should be funded related to the impact of COVID-19 [9].

## The current challenge

Most mental health systems across the world have been woefully inadequately funded, planned, organised and delivered given the major global burden of mental disorders [10]. The CODID-19 pandemic has added even greater challenges. With shrinking economies, policy makers will have to rebalance prioritizing mental health services against other health service investments. The ability to react and take appropriate decisions will depend on the existing resources and infrastructure. These decisions will then need to be matched up against the impact of the pandemic—not only on mental health, but to the overall health of the country, as well as the socioeconomic determinants. Thus, it becomes important to have a better understanding of what steps can be taken in such scenarios to make most efficient use of the limited resources. At the same time new research should align with the changing paradigm of mental health care delivery which may have to rely on use of digital solutions [11], identify risk factors that are particularly relevant to precipitating mental disorders in the face of this pandemic, and develop and implement scalable interventions to mitigate the impact of the infection on mental health across different communities and different settings.

In this context, the aim of this paper is to outline a roadmap to guide countries to strengthen mental health systems to tackle the increasing burden of mental disorders. Using both World Health Organization’s Mental Health Action Plan 2013–2020 [12] and the WHO health systems strengthening framework [13], we propose a set of recommendations from the perspectives of policy makers, service providers and research funders, organised into low-, middle- and high-resource scenarios. While the recommendations encompass systematic and structural actions that are relevant to building a strong mental health system per se and is essential to the current pandemic as in any other crisis, embedded within them are some more specific aspects that are particularly relevant to the COVID crisis, and these have been indicated separately. The eventual objective is to “build back better” [14].

## Recommendations to overcome the challenge

Table 1 shows recommendations for policy makers in areas of leadership and governance, finance, policies and programmes that include long term care and needs of vulnerable populations.

**Table 1:** Mental health system specific recommendations relevant to policy maker by available resources during the pandemic

Recommendations relevant to policy makers	Low resource settings with few mental health resources and infrastructure	Medium resource settings with some mental health resources and infrastructure (includes recommendations in addition to those in low resource settings)	High resource settings with good mental health resources and infrastructure (includes recommendations in addition to those in low and medium resource settings)
Strengthen leadership and governance	* Using an intersectoral approach, develop evidence-based policies to manage basic mental health needs, and strategies for implementation of those policies, especially involving teams managing COVID infection *Inform and support politicians, and administrators to the need of mental health interventions in the light of this pandemic using existing evidence-based communication tools available on the internet *Have clear standard operating procedures (SOPs) to operationalize implementation strategies which are well integrated within the larger framework to tackle COVID infection *Develop and implement monitoring mechanisms using feasible indicators to capture prevalence of common mental disorders, number of mental health service providers and accessibility Mental health care is integrated within the overall health and humanitarian policy; decentralize the administrative machinery but ensure that certain sound principles are adhered to by all	*Mental health needs of special vulnerable population groups addressed using appropriate policies and programmes and these should focus on the poor and marginalized communities *Existing policies and programmes are tailor made to specific regional needs even within a country based on data about the burden of mental disorders in the community *Special training of policy makers are undertaken to increase knowledge about mental health in the face of this pandemic by mental health professionals	*Key aspects of the policies are geared around inclusivity and equity and specifically target vulnerable populations of different types; Teams and departments in charge of each component are outlined and accountability is addressed appropriately using suitable indicators; data collection is synchronized within the larger system that gathers data on the pandemic
Identify appropriate finance mechanism to support policies and programmes; develop schemes to cover longer term mental health care	*There are earmark additional funds to cover evidence-based mental health activities under health budget at national, regional and district level; especially ring-fence budget to cover the mental health impact due to COVID pandemic *Mental health expenditure is tracked and protected and mechanisms are created to do so if not in place, given increased proportion of health budget allocation for COVID *Include mental health within the care packages of social and private insurances at least to cover expenses arising out of the pandemic; identify micro financing schemes to support mental health support for individuals who may need extensive mental health care	*Budget allocated for mental health care during this crisis can be spread across different intersectoral activities including poverty alleviation programmes, employment generation activities, where addressing mental health needs is prioritised in those programmes; funds should look at both short and long term outcomes A large pool of private and public insurance schemes is in place to cover mental health care costs	*Mental health budget to address enhanced needs of mental health burden during the pandemic is increased significantly to cover current and future needs and those are tracked using robust systems *Private or public insurance systems have mental health care integrated within their policies and is adequate to cover for long term care and included in packages to cover COVID
Promote programmes specifically targeting vulnerable groups	*Identify specific components within other existing programmes such as those targeting homelessness, employment, migrant populations which focus on mental health needs of such populations, especially given the impact of the pandemic on these populations *Develop mental health promotion communications focused on stigma related to the pandemic and its effect on mental health If any mental health program exists, then strengthen those to specifically address needs during the crisis; specifically ensure that such programmes are accessible to those needing it Programmes are culturally relevant and tested; but programmes tested in similar settings but in some other areas may be used in order to save time and money	*Develop new mental health programmes that are not only focused on addressing the immediate mental health needs due to the COVID pandemic but has long term benefits too; implement those programmes amongst populations in need- migrants, women, elderly, children *Involve relevant stakeholders (government or non-government) in the programmes being developed and target programmes to cater to the specific needs of individuals, families, care givers, employers, different vulnerable groups *Develop targeted programmes for local settings which specifically focus on issues such as domestic violence, child abuse, elderly care, alcohol and substance use, reducing suicide risk which are showing increased prevalence during this pandemic	*Enhance any existing programmes that already focus on vulnerable populations Integrate mental health programmes within other social sector specific programmes such as those on housing, employment generation, elderly care, child care, school systems

*Recommendations that are particularly relevant for managing mental health issues during COVID infection

Table 2 outlines recommendations for service providers and other stakeholders involved in care of those with mental health problems. It focuses on providing equitable and accessible community-based mental health services and clinic-based services for those needing such care, build capacity by training primary care health workers to provide community-based services, implement community-based mental health prevention and promotion programmes, strengthen civil societies to support the government mental health service provision, and support programmes and policies specifically to manage workplace related stress which will be a major issue given the economic woes and changing paradigms of limited workforce or working from home.

**Table 2:** Recommendations relevant to service providers and other stakeholders by available resources during the pandemic

Recommendations relevant service providers and other stakeholders	Low resource settings with few mental health resources and infrastructure	Medium resource settings with some mental health resources and infrastructure (includes recommendations in addition to those in low resource settings)	High resource settings with good mental health resources and infrastructure (includes recommendations in addition to those in low and medium resource settings)
Develop or strengthen equitable, accessible and appropriate community-based mental health services and clinic-based services for those needed special care	*Enable communities to identify common mental disorders and severe mental disorders; develop routine mechanisms to collect such data during the pandemic *Make care provision inclusive especially for those most affected such as migrants, women, elderly populations, children and adolescents, who may be affected more during this pandemic *Connect community, primary, secondary and tertiary care systems using innovative care models that are accessible and equitable; secondary and tertiary level should be able to provide care to those with more severe mental health problems *Telemedicine using integrated health information systems is actively pursued to ensure remote monitoring and followup. *Robust supply chains are in place to ensure availability of all psychotropic medications listed on a countries’ Essential Drugs List; this becomes more important during the pandemic as accessibility and movement is restricted thus compromising ability to purchase medicines from private pharmacies which may be few especially in rural communities The care model centres around protection of human rights	*Upgrade the primary and secondary health system to manage mental health problems; have provisions for basic counselling at primary level; enhance community-based services to support those needing support for daily living such as groceries, laundry, etc. *Address specific needs of populations who may be particularly affected such as women facing domestic violence, children or adolescents facing abuse, individuals at high risk of suicide or self-harm, those with substance use disorders, elderly with dementia *Secondary care level has the capacity to manage complicated cases with multimorbidity which would be more common among those infected with COVID19, only the more severe cases are referred to tertiary level; electronic medical records systems are enabled for appropriate follow-up *The health information system allows patients to routinely track symptoms and link them to the medical records so that health care providers can track them easily and prioritize followup Specific programmes for those needing institutional care or elderly support are not compromised	* Integrate mental health services within care for COVID infection at each level *Health information systems are linked across different health conditions so that health providers can easily track multiple conditions and the patient can tack their health too Support is available for carers of persons with mental illnesses Care for special population such as elderly, child and adolescents are ensured Multiple medication options are available with clinicians to treat complicated cases Health system has the capacity to reach out to low or medium resource settings and enhance their capacities to provide human rights centred care
Train adequate primary care staff to cater to the increased mental health needs at the community level	*Awareness is provided about stigma related to mental disorders using multimedia strategies, with a specific focus on the increased risk of stigma associated with COVID infection *Train primary health workers on mhGAP and especially focus on the modules of depression, emotional stress, alcohol use disorders, suicide risk; primary care doctors should be able to identify psychotic symptoms and manage common mental disorders efficiently; fear or anxiety related to infection and death and bereavement should be managed through basic counselling skills	*Psychological therapies are more advanced than basic counselling to manage mental health issues related to COVID infection and overcoming death of loved ones Primary health workers are trained on the full mhGAP and manage most cases as per guidelines; linkages with mental health professionals should be enabled using e-health or m-health platforms Community based programmes to raise awareness about mental health and reduce stigma are implemented by health staff and other stakeholders	*Stigma campaigns are tailored to specific communities such as young or old, jobless, men or women, schools, workplace; campaigns should specifically address stigma associated with COVID infection Primary care staff supported by paramedical staff including ambulance services can identify and care for most mental disorders outlined under mhGAP; support from trained mental health professionals are sought as needed
Implement mental health promotion and prevention programmes	*Evidence-based and preferably culturally relevant prevention and promotion programmes to reduce stigma, raise awareness about common mental disorders and need for addressing mental health needs arising out of the COVID crisis are implemented across different settings to the extent feasible * Even if the programmes are basic in structure they should be implemented as widely as possible, and all health promotion activities are based on sound behaviour science principles; programmes are integrated as much possible within other programmes addressing COVID infections Government backed pan-regional programmes with support from local non-governmental partners are implemented	*Culturally relevant programmes addressing stigma for special populations such as health workers, child and adolescents, women are developed and implemented Non-governmental organizations partner with government agencies to implement programmes in different settings and population groups Programmes are developed for special settings like factories, prisons, schools that need different implementation pathways	*Mental health needs of special populations such as elderly, prisoners, women, LGBTQIA communities, institutionalized communities, poor and marginalized migrant workers, refugees, etc., are specifically tailored and include measures on how to address specific issues of COVID 19 related mental illness Suitable programmes are available for families and carers of persons with mental illnesses
Strengthen civil societies	*Civil societies identify key areas where they can contribute and pitch into support the overall government plan to manage mental health problems during the COVID pandemic *Civil societies involved in mental health service delivery or research or advocacy are identified and integrated within a government database; especially those with the ability to support multiple health conditions including mental health should would be beneficial The databases of civil societies allow the administrators to identify strengths of each organization, its reach, focus, and key resource person(s) Government plans their mental health alleviation programmes keeping civil societies in the loop and takes their opinions Government allocates ring-fenced funds to support activities undertaken by civil societies where it by itself cannot function effectively, be it research, program implementation, or advocacy	*A registry of civil societies is advanced enough to allow for an easy two-way communication between them and the government *Appropriate funds to support civil societies led programmes are present and those are planned in consultation with the government Civil societies per se can access resources and roll out programmes as per their strengths while keeping the overall focus on managing the impact of the pandemic The collaborations between civil societies and government is streamlined; the government provides oversight to local and regional programmes that are essentially implemented by civil societies	*Civil societies and government are equal partners in delivering care or conducting research during this pandemic *Civil societies working at national, regional or local levels are adequately funded to support not only their own activities but support government efforts to overcome the COVID pandemic
Enable employers to manage stress at workplace	*Suitable communication packages are present to engage with employers to discuss workplace stress, COVID related mental health issues, stigma *Initiate a dialogue with different employer’s associations to understand specific challenges related to COVID and how they can affect mental health *Specific interactions with employers in health care sectors, police, civil administrations who are at the forefront of managing the crisis are initiated; programmes are in place to address fear and anxiety related to being infected or death of loved ones *Labour laws are revisited or redrafted to ensure employee welfare during this pandemic	*Interaction with employer’s associations are better integrated and builds on existing models of such interactions with specific focus on managing the pandemic	*Specific programmes are developed to support needs of niche employers who are at the forefront of managing the COVID crisis, and these need to be tailored to local needs Welfare programmes for employers focused on reducing stress are in place and the staff are actively engaged in those; employers also help the programmes to grow organically through their active inputs

*Recommendations that are particularly relevant for managing mental health issues during COVID infection

Table 3 outlines recommendations for researchers and research funders to align research to strengthen information systems, gather more epidemiological data and conduct robust interdisciplinary interventions that are scalable, use innovative designs and leverage technology to develop some interventions to facilitate service delivery and improve supply chain of psychotropic medications at primary care levels and leverage the power of social media to deliver interventions. Technology led solutions need to be ramped up especially in these conditions where in-person data collection is limited considerably.

**Table 3:** Recommendations relevant to researchers and research funders by available resources during the pandemic

Recommendations relevant researchers and research funders	Low resource settings with few mental health resources and infrastructure	Medium resource settings with some mental health resources and infrastructure (includes recommendations in addition to those in low resource settings)	High resource settings with good mental health resources and infrastructure (includes recommendations in addition to those in low and medium resource settings)
Develop research to improve information systems	*Identify key mental health indicators that can be tracked over time through government applications/websites, and are built into the tracking system for COVID infection; non-governmental organizations too to include such indicators in their activities *Enable community health workers with simple questionnaires to capture stressful risk factors, and common symptoms pertaining to any mental disorders based on the mhGAP screening application or other similar tool *Develop a website for such data to be automatically uploaded and analysed	*Besides basic mental health related data, more details are gathered around access and use of mental health services and stigma perceptions in the face of the pandemic *The health websites should enable automated data analyses and report generation filtered by predefined criteria	*Health information systems are linked to detailed clinical data related to COVID infection and other risk factors *The system allows triangulation of data from different sources and linking with other data to provide a rich matrix of data to do sophisticated analyses using machine learning
Develop research on epidemiology, neurobiological effects, community-based and special population-based interventions, linkages with environmental and social sciences	*Conduct research to understand the epidemiology of mental health impact of the COVID pandemic across different population groups, especially those at increased risk; understand correlated factors and account for non-health related factors that are also impacting mental health Develop interventions to manage the mental health impact of the pandemic using community-based approaches based on implementation science principles Ascertain factors that determine adequate mental health service use during and after the current pandemic is over	*More resource intensive research conducted that covers larger geographical areas and provides more robust data both from an epidemiology and scalable intervention perspective, including those involving vulnerable populations *Larger randomized studies are implemented to generate robust data that will not only benefit current knowledge related to the impact of COVID infection on mental health but also inform future programmes and policies *Existing electronic databases allow intersectoral research to assess multiple dimensions related to the pandemic that affect mental health *Use social media usage analytics to assess stress and mental illness using machine learning tools Research focuses on understanding treatment outcomes to specific medications and its correlation to mental health	*Neurobiological research into the impact of COVID 19 on brain including early childhood development and brain neuroplasticity should be planned *Behavioural research explores mental health impact of the pandemic due to physical distancing, loneliness, stigma, poverty, hunger, among other issues *Machine learning and research involving artificial intelligence uses correlated datasets and modelling to forecast mental health impact of the pandemic; research has implications for future predictive models too Longer term effects of the stress on human psychology can be conducted and interventions to mitigate such can be implemented
Develop innovative solutions to improve mental health systems; support technology-enabled solutions to support service delivery; identify strategies to enable more efficient supply chain logistics models for medicines; use of social media to deliver interventions on mental health promotion	*Develop technology-enabled solutions to conduct research and gather data avoiding in-person contact as much as feasible, while ensuring appropriate data security and privacy *Identify culturally relevant evidence-based applications to gather data on mental health outcomes and increase access to care Conduct health systems research to investigate how supply of psychotropic medications at community level can be accomplished Use social media platforms to not only link researchers but also develop interventions based on use of social media	*Better ability to link secondary data from other sources with primary data using big data analytics	*Service use involves digital technology, interactive voice messages, video games, virtual reality *Advanced methodologies using artificial intelligence driven analytics allow development of risk profiles in real time and identify predictive models

*Recommendations that are particularly relevant for managing mental health related problems during COVID infection

## Discussion

Mental health is one of the most neglected areas of health. The COVID 19 pandemic and any similar challenges in future, should be tackled along the lines of a humanitarian emergency [15]. Even during more normal times, addressing mental health needs as part of the Sustainable Development Goals has been a major challenge [6]. The COVID crisis has led to a fragmentation of existing health systems across the globe, which will have a profoundly negative and cascading effect on mental health not only in coming months, but for some years, and this has been identified even at the United Nations [2]. Not only will COVID 19 lead to a surge in mental health needs in the community [5, 7], but the way it has crippled the health systems globally to address the need of any other health problem, it is likely to have a devastating effect on the longer term needs of people who need care for mental illnesses [5].

### Strengthening existing mental health systems to tackle the pandemic

It becomes necessary to identify strategies to strengthen health systems to overcome these challenges. The best way to tackle mental health impact is to not limit it to overcoming the immediate mental health crisis, but to embed its management within the larger health system that can impact the lives of individuals globally or across large regions. In this paper we have focused on low, middle and high resource settings and indicated how they can re-orient their health systems, service provision and research according to the need and available resources. This approach applies as much to countries as it does to regions or health administrative units within countries, given the very large disparities in needs and resources that are common in countries worldwide. We present the recommendations in Tables 1, 2 and 3 not as separate and unrelated proposals, but as part of an overall integrated approach to health system strengthening, which should be adapted to specific local needs and modified in relation to available resources.

### Recommendations for policy makers

Policy makers will play a major role in providing leadership to any programmes and policies that they develop and implement. It is therefore imperative that they are both educated about the mental health needs during this crisis and supported by academicians and mental health professionals to develop robust policies and programmes to address the increased burden of mental health. While there will be a requirement to address some immediate mental health needs and provide psychosocial support in line with the IASC guidelines [15], they should plan on developing more robust policies and programmes to build a system that is more holistic, encompasses intersectoral collaborations, protects the rights of the individuals, has deliverables that are based on evidence, and is able to deliver care over a long time.

These policies and programmes should be supported by adequate funding and tap into existing private and government sources. Insurance mechanisms should ensure that adequate financial support is available for individuals to seek mental health care as per need. This may need a paradigm shift in the way the insurance system is organized as most often mental disorders are excluded from their remit. In United States of America, telehealth parity has been introduced in many other states post the COVID crisis to ensure providers get same payment for teleconsultations as in-person consultations, thus enabling service delivery [16]. Telepsychiatry has also resulted in expanding home-based care for conditions like substance use disorders in the United State, which earlier were only available if comorbid physical disorders were present. Policy makers should support development of teleconsultations and robust electronic health records systems to enable remote care delivery.

The mental health budget allocation should reflect the change in the burden due to the crisis and the government should be open to exploring innovative ways to build in mental health related budget into the relevant sectors, for example, addressing job security, providing affordable homes for migrant workers, building shelters for women or children facing abuse, enhancing care for the elderly and those with dementia, could help in reducing the burden considerably. Strategies should be locally relevant and keep needs of vulnerable populations, inclusivity, stigma reduction, and rights-based approaches at the core of their principles [12].

### Recommendations for service providers and other stakeholders

The key elements that service providers should keep in mind are to develop a model that is community-based and involves training and upskilling of primary health workers and non-mental health professionals to both identify and deliver basic mental health care based on principles laid down by existing guidelines [12], and drawing on basic tenets and the detailed guidance of the mhGAP programme of the World Health Organization. Psychological therapies can be tailored to the level of skilled resources available.

The level of specialized care provided should be informed by local factors and available resources. Some of those are number of mental health trained staff and their skills level, types of mental health facilities available, for example primary, secondary or tertiary care, budgets available to support services, availability of community-based support services to cater to specific needs of individuals with significant disabilities, support services for families and caregivers, role of multi-sectoral agencies to support mental health care such as employment agencies, housing, elderly welfare, child welfare services, education. Services provided should be locally tested and culturally relevant. Needs of vulnerable populations should be specially kept in mind. The services should be both accessible and equitable, and one key strategy to ensure that in times of physical distancing could be increased use of technology enabled services such as e-health, m-health, telemedicine [11, 17]. This should encompass screening, service delivery, training of health workers and monitoring.

A key aspect is to maintain physical distancing while ensuring continuity of care. To do so telemedicine services and linking of patient and provider data on health information systems that enables tracking of a patient’s health remotely is necessary. The system should allow both the patient and health providers to interact with each other either through video chats or dedicated phone lines and be interactive enough to allow the patient to upload their progress, treatment adherence and complications online and the provider can respond to those in real time. Reports from Italy, underline how mental health services were prioritized in the face of the COVID pandemic by identifying essential mental health services, providing medications to those with substance use disorders, enabling teleconsultations [18, 19]. Even in low resource settings such as In India, teleconsultation for mental health issues is being regularly provided by many tertiary care centres, though there is a lot of scope for improvement. Civil societies have also set up teleconsultation to care for emergency situations [20]. In China, there were more specific challenges as being the first country to face the pandemic, there were no prior experiences to follow, but restructuring of service at different levels and delivering a mix of online and offline services were identified as critical for ensuring continuity of care, but new ethical challenges related to teleconsultations and practical problems related to implementation of new strategies had to be overcome [21].

Availability of psychotropic medicines should be facilitated by ensuring that the supply-chain is maintained, and governments need to invest for that specifically in low and middle resource settings. Civil societies should be encouraged to collaborate with government agencies and work in both strategizing and service delivery and the government should allocate ring-fenced funds for such activities. Labour organizations and employers should be adequately trained to identify specific mental health needs of individuals in this pandemic, but also encouraged to revisit their policies to ensure that their laws are employer friendly but also allowing for industry growth. Addressing the mental health needs of employees is critical even in normal times [22] and during this added challenge it may be a major factor to alleviate the burden as employees and employers both grapple with new situations of working from home, restricted office attendance, staff layoff, reduced productivity, and reduced remunerations.

### Recommendations for researchers and research funders

The focus of research and the level of sophistication of such will vary across low, medium and high resource settings. Even within a high-income country there may be a need to understand how to deliver basic services or ascertain prevalence or incidence of mental disorders in some regions with lower resources. In order to capture the true burden of COVID 19 on mental health, it is vital that information systems to gather such data is strengthened across all settings. It is important to create a system where data from multiple sources can be linked to build an aggregate database involving both clinical and social determinants. An initiative on this, Countdown Global Mental Health 2030 is already underway [23]. Research exploring neurobiological correlates, behavioural concepts that determine how stigma and discrimination plays a role in help seeking in COVID affected individuals, effect of socioeconomic policies on mental health, mental health effects on different populations by age groups, gender, migrant and labourer communities, homeless, health workers, etc., are all relevant areas of further investigation [9, 24, 25]. Research should also explore newer strategies using machine learning and artificial intelligence to build predictive models to inform risk profiles for future pandemics and determine possible phenotypes that could allow service providers to modulate care and overall outcomes. The role of artificial intelligence, digital tools to collect real-time data, combining online and off-line data with in-person data needs to be enabled to enrich research data to support better care models [26].

## Conclusion

We believe that urgent action is needed to strengthen mental health system in all settings in view of enhanced need for mental health care and decreased access during and beyond the COVID-19 pandemic. The roadmap draws upon key sources and accumulated knowledge of mental health systems globally to provide a perspective on practical steps to strengthen mental health systems across the world. The strategies outlined here can be used as a guide to develop these further or identify new ones that are more applicable to local settings. Taking no action in the face of increasing threats to mental health of populations is not an option in the COVID era. The roadmap that we recommend here is intended to be used as a guide by policy makers, service providers and other stakeholders, researchers and research funders to develop strategies to actively improve mental health in relation to COVID 19 following the principle of building back better [14] and deliberations of the National Academy of Sciences where suggestions were made to have person centred care, shared decision making and patient and family engagement [27], and to make mental health an integral part of the management of COVID 19 [28].
